# Clinical characteristics, prognostic factors, and survival trends in esophageal neuroendocrine carcinomas: A population‐based study

**DOI:** 10.1002/cam4.4829

**Published:** 2022-05-21

**Authors:** Chuyan Chen, Haiyi Hu, Zhibo Zheng, Yi Yang, Wei Chen, Xinwei Qiao, Peng Li, Shutian Zhang

**Affiliations:** ^1^ Department of Gastroenterology, Beijing Friendship Hospital, Beijing Key Laboratory for Precancerous Lesion of Digestive Disease National Clinical Research Center for Digestive Disease, Beijing Digestive Disease Center, Capital Medical University Beijing China; ^2^ Department of International Medical Services Peking Union Medical College Hospital, Chinese Academy of Medical Sciences Beijing China; ^3^ Department of Thoracic Surgery Peking Union Medical College Hospital, Chinese Academy of Medical Sciences Beijing China

**Keywords:** esophageal, neuroendocrine carcinoma, prognostic factors, SEER, survival

## Abstract

**Background:**

Esophageal neuroendocrine carcinoma (ENEC) is an extremely rare type of malignancy. Clinical data of ENEC are limited to case reports and case series. More information is needed on its clinical feature, management, and prognosis.

**Methods:**

This study collected information of ENEC patients diagnosed pathologically from 2010 to 2018. Data including demographic information, clinical features, and survival trends were obtained from the Surveillance, Epidemiology, and End Results (SEER) database. Statistical analyses were performed with STATA/SE 15.1, SPSS 25.0, and GraphPad Prism 8.

**Results:**

A total of 283 ENEC patients were included in this study. The small‐cell and large‐cell subtypes of ENEC possess similar clinical features. The lower third of the esophagus (58%) was the most common location of ENEC. At the time of diagnosis, most ENEC patients were AJCC 7th stage IV (48.1%). Metastasis occurred in more than half of the ENEC patients (53.4%), and the most common metastatic site was the liver (37.1%). Compared with poorly differentiated esophageal squamous cell carcinoma (ESCC), another aggressive malignancy of the esophagus sometimes confused with ENEC because of similar histological features, our study showed differences in tumor location and metastatic rate, but similar poor survival rates. Multivariate survival analysis showed that ENEC located at the middle third of esophagus (*p* = 0.013), “Brain metastasis” (*p* = 0.019), and “Liver metastasis” (*p* < 0.001) were independent predictors of worse outcomes. “Surgery” (*p* = 0.003), and “Chemotherapy” (*p* < 0.001) were associated with better survival.

**Conclusion:**

A significant proportion of patients with newly diagnosed ENEC presented with metastatic disease. Predictors of poor survival included tumor location, brain metastasis, and liver metastasis. ENEC and poorly differentiated ESCC share certain histological features, but differ in tumor location and metastatic rate. Yet, no standard treatment strategy has been established, but surgery and chemotherapy were related to better outcomes.

## Graphical abstract

Esophageal neuroendocrine carcinoma (ENEC) is an extremely rare type of malignancy. This study collected information of 283 ENEC patients diagnosed pathologically from 2010‐2018. Multivariate survival analysis showed that ENEC located at the middle third of esophagus (*p* = 0.013), “Brain metastasis” (*p* = 0.019), and “Liver metastasis” (*p* < 0.001) were independent predictors of worse outcomes. “Surgery” (*p* = 0.003), and “Chemotherapy” (*p* < 0.001) were associated with better survival.

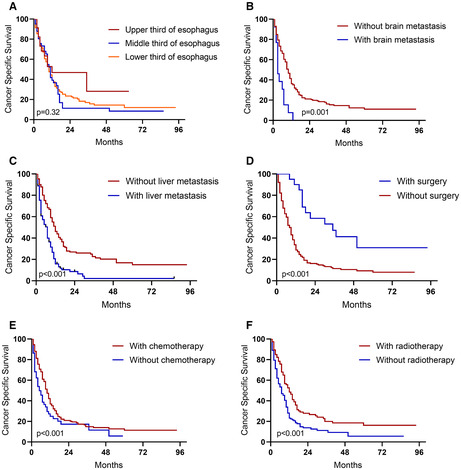

## 1 INTRODUCTION

Esophageal neuroendocrine carcinoma (ENEC) is an extremely rare type of malignancy, accounting for only 0.04–4.6% of all gastroenteropancreatic neuroendocrine tumors,^1^ and approximately 0.4–2% of all esophageal neoplasms.^1^, ^2^, ^3^, ^4^, ^5^, ^6^ Due to high malignant potential and poor prognosis of ENECs, our understanding of this malignancy is mainly based on case reports and case series.^7^, ^8^, ^9^

The prevalence of ENEC has gradually risen over the past 10 years,^10^ but clinicians still lack treatment experience and suitable guidelines. A systematic description of its epidemiology feature, tumor characteristic, treatment strategy, and prognosis is needed. Therefore, in this study, we utilized information of ENEC patients extracted from the publicly available Surveillance, Epidemiology, and End Results (SEER) database, and aimed to analyze the clinicopathological characteristics of ENECs and identify those that affected prognosis.

Histologically, ENEC can be divided into two subtypes, the small‐ and large‐cell NEC. In the past, most studies have focused on small‐cell NEC, but large cell carcinoma also accounts for an important part of esophageal NEC. Whether large‐cell NEC exhibits different clinicopathological features from small cell NEC remains to be further investigated. In this study, we also hope to utilize data from the SEER database to find out whether esophageal large‐cell NEC should be treated differently.

Poorly differentiated squamous cell carcinoma (SCC) is another aggressive malignancy of the esophagus. Some histological and clinical features of ENEC frequently overlap with poorly differentiated SCC, leading to a high rate of misdiagnosis, especially for small biopsy tissue samples.^11^ In addition, recent studies have proposed that there are a number of similarities in biological and molecular features between the neuroendocrine component and the non‐neuroendocrine component of gastrointestinal NECs.^12^, ^13^ Another group even proposed that ENEC might originate from SCC cells, due to almost absence of neuroendocrine cells in normal human esophagus.^14^ Recognizing these associations between ENEC and esophagus SCC (ESCC) is important to help us understand the carcinogenesis of NEC, and to guide our treatment. Therefore, we also sought to explore the similarities and differences in clinicopathological features between ENEC and poorly differentiated ESCC.

## 2 METHODS

### 2.1 Study population and data collection

The information of ENEC and ESCC patients diagnosed between 2010 and 2018 according to the International Classification of Disease (ICD) were extracted from the database of Incidence‐SEER 18 Custom Data (with additional treatment fields) using SEER*Stat version 8.3.5. ENEC and ESCC were defined according to the ICD for oncology version 3 (ICD‐O‐3). Site recode ICD‐O‐3/2008 referring to the esophagus was used to define the primary site, and histological codes 8013/3, 8041/3, and 8246/3 representing large cell neuroendocrine carcinoma, small cell carcinoma, and neuroendocrine carcinoma not otherwise specified respectively were included for ENECs. Morphology code 8070/3 was used to identify ESCC patients. Demographic variables included gender, age, race (white, black, and others), and marital status. The TNM stage according to the seventh edition criteria of the American Joint Committee on Cancer (AJCC) staging system, as well as clinical features included tumor location (upper third, middle third, lower third of the esophagus, or unspecific), metastasis site, therapeutic methods, and survival data were obtained for analysis.

### 2.2 Statistical analysis

Patient age was converted to a categorical variable (<65 and ≥65) for analysis considering the distribution of it in the cohort. The Chi square test was used to compare categorical variables among different groups. Relative impacts of risk factors were analyzed using the univariate and multivariate Cox proportional hazard models. Kaplan–Meier survival curves were plotted to analyze cancer‐specific survival, and survival analysis was carried out using the log‐rank test. Statistical analyses were conducted with STATA/SE 15.1 (StataCorp, 2017), SPSS 25.0 (SPSS Inc., Chicago, IL, USA), and GraphPad Prism 8 (GraphPad Software, CA, USA). *p* < 0.05 was considered statistically significant.

## 3 RESULTS

### 3.1 Clinical features of different subtypes of ENEC patients

Table 1 shows clinical features of the two subtypes of ENEC and neuroendocrine carcinoma not otherwise specified. Only sex (*p* = 0.02) and location of tumor (*p* = 0.011) showed significant difference on comparison. ENECs are mainly located in the lower third of the esophagus, and this feature is more pronounced in large cell NECs (71.9%). Regional and distal metastasis, as well as metastatic site showed no difference among groups. Also, 2‐ and 5‐years disease‐specific survival appeared to be similar between different subtypes.

**TABLE 1:** Clinical features of different subtypes of esophageal neuroendocrine carcinoma patients

Characteristics	Large cell NEC *N* = 32(%)	Small cell carcinoma *N* = 129 (%)	Neuroendocrine carcinoma *N* = 122 (%)	*p* value
Sex				0.020
Male	26 (81.3)	87 (67.4)	100 (82.0)	
Female	6 (18.8)	42 (32.6)	22 (18.0)	
Age				0.515
<65	14 (43.8)	51 (39.5)	57 (46.7)	
≥65	18 (56.3)	78 (60.5)	65 (53.3)	
Race				0.255
White	29 (90.6)	101 (78.3)	105 (86.1)	
Black	2 (6.3)	14 (10.9)	6 (4.9)	
Other	1 (3.1)	14 (10.9)	11 (9.0)	
Marital status				0.321
Married	20 (62.5)	73 (56.6)	61 (50.0)	
Singled	11 (34.4)	45 (34.9)	55 (45.1)	
Unknown	1 (3.1)	11 (8.5)	6 (4.9)	
Location				0.011
Upper third of esophagus	1 (3.1)	15 (11.6)	8 (6.6)	
Middle third of esophagus	0	30 (23.3)	22 (18.0)	
Lower third of esophagus	23 (71.9)	64 (49.6)	77 (63.1)	
Unspecific	8 (25.0)	20 (15.5)	15 (12.3)	
T‐stage				0.852
T1	8 (25.0)	26 (20.2)	27 (22.1)	
T2	2 (6.3)	5 (3.9)	9 (7.4)	
T3	8 (25.0)	22 (17.1)	23 (18.9)	
T4	4 (12.5)	20 (15.5)	15 (12.3)	
Unspecific	10 (31.3)	56 (43.4)	48 (39.3)	
N‐stage				0.552
N0	10 (31.3)	37 (28.7)	26 (21.3)	
N1	18 (56.3)	61 (47.3)	59 (48.4)	
N2	1 (3.1)	9 (7.0)	9 (7.4)	
N3	1 (3.1)	3 (2.3)	7 (5.7)	
Unspecific	2 (6.3)	19 (14.7)	21 (17.2)	
M‐stage				0.650
M0	11 (34.4)	60 (46.5)	57 (46.7)	
M1	21 (65.6)	67 (51.9)	63 (51.6)	
Unspecific	0	2 (1.6)	2 (1.6)	
TNM‐stage				0.367
I	1 (3.1)	7 (5.4)	5 (4.1)	
II	4 (12.5)	10 (7.8)	15 (12.3)	
III	1 (3.1)	19 (14.7)	18 (14.8)	
IV	21 (65.6)	58 (45.0)	57 (46.7)	
Unspecific	5 (15.6)	35 (27.1)	27 (22.1)	
SEER stage				0.235
Localized	4 (12.5)	13 (10.1)	9 (7.4)	
Regional	6 (18.8)	24 (18.6)	36 (29.5)	
Distant	22 (68.8)	81 (62.8)	68 (55.7)	
Unspecific	0	11 (8.5)	9 (7.4)	
Bone metastasis				0.333
No	25 (78.1)	107 (82.9)	102 (83.6)	
Yes	7 (21.9)	17 (13.2)	13 (10.7)	
Unspecific	0	5 (3.9)	7 (5.7)	
Brain metastasis				0.521
No	29 (90.6)	116 (89.9)	108 (88.5)	
Yes	3 (9.4)	6 (4.7)	6 (4.9)	
Unspecific	0	7 (5.4)	9 (6.6)	
Liver metastasis				0.717
No	19 (59.4)	74 (57.4)	72 (59.0)	
Yes	13 (40.6)	49 (38.0)	43 (35.2)	
Unspecific	0	6 (4.7)	7 (5.7)	
Lung metastasis				0.520
No	28 (87.5)	110 (85.3)	98 (80.3)	
Yes	4 (12.5)	12 (9.3)	17 (13.9)	
Unspecific	0	7 (5.4)	7 (5.7)	
Surgery				0.399
No	28 (87.5)	120 (93.0)	108 (88.5)	
Yes	4 (12.5)	9 (7.0)	14 (11.5)	
Chemotherapy				0.466
No	6 (18.8)	37 (28.7)	36 (29.5)	
Yes	26 (81.3)	92 (71.3)	86 (70.5)	
Radiotherapy				0.960
No	18 (56.3)	75 (58.1)	72 (59.0)	
Yes	14 (43.8)	54 (41.9)	50 (41.0)	
Survival				0.921
2‐year disease specific survival	19.6%	18.8%	17.3%	
5‐year disease specific survival	0	8.4%	13.8%	

### 3.2 Characteristics of ENEC patients and comparison between ENEC and poorly differentiated ESCC

Table 2 the shows clinical characteristics of the ENEC patients and the comparison results against poorly differentiated ESCC patients. A total of 283 ENEC patients and 2043 poorly differentiated ESCC patients from 2010 to 2018 were included in our study. Most patients in both groups were diagnosed at the age of more than 65 years. ENEC was more likely to occur in married (54.4%), white (83.0%), and male (75.3%) people. The lower third of the esophagus (58%) was the most common location of ENECs, which significantly differs from poorly differentiated ESCC (*p* < 0.001).

**TABLE 2:** Clinical characteristics of esophageal neuroendocrine carcinoma and poorly differentiated squamous cell carcinoma patients

Clinical characteristics	Esophageal neuroendocrine carcinoma *N* = 283 (%)	Poorly differentiated squamous cell carcinoma *N* = 2043(%)	*p* value
Sex			0.014
Male	213 (75.3)	1391 (68.1)	
Female	70 (24.7)	652 (31.9)	
Age			0.758
<65	122 (43.1)	861 (42.1)	
≥65	161 (56.9)	1182 (57.9)	
Race			<0.001
White	235 (83.0)	1331 (65.1)	
Black	22 (7.8)	475 (23.3)	
Other	26 (9.2)	237 (11.6)	
Marital status			<0.001
Married	154 (54.4)	875 (42.8)	
Singled	111 (39.2)	1054 (51.6)	
Unknown	18 (6.4)	114 (5.6)	
Location			<0.001
Upper third of esophagus	24 (8.5)	468 (22.9)	
Middle third of esophagus	52 (18.4)	635 (31.1)	
Lower third of esophagus	164 (58.0)	598 (29.3)	
Unspecific	43 (15.2)	342 (16.7)	
T‐stage			<0.001
T1	61 (21.6)	377 (18.5)	
T2	16 (5.7)	162 (7.9)	
T3	53 (18.7)	564 (27.6)	
T4	39 (13.8)	333 (16.3)	
Unspecific	114 (40.3)	607 (29.7)	
N‐stage			0.004
N0	73 (25.8)	683 (33.4)	
N1	138 (48.8)	830 (40.6)	
N2	19 (6.7)	217 (10.6)	
N3	11 (3.9)	85 (4.2)	
Unspecific	42 (14.8)	228 (11.2)	
M‐stage			<0.001
M0	128 (45.2)	1338 (65.5)	
M1	151 (53.4)	689 (33.7)	
Unspecific	4 (1.4)	16 (0.8)	
TNM‐stage			<0.001
I	13 (4.6)	164 (8.0)	
II	29 (10.2)	309 (15.1)	
III	38 (13.4)	558 (27.3)	
IV	136 (48.1)	689 (33.7)	
Unspecific	67 (23.7)	323 (15.8)	
Bone metastasis			0.045
No	234 (82.7)	1768 (86.5)	
Yes	37 (13.1)	175 (8.6)	
Unspecific	12 (4.2)	100 (4.9)	
Brain metastasis			<0.001
No	253 (89.4)	1910 (93.5)	
Yes	15 (5.3)	23 (1.1)	
Unspecific	15 (5.3)	110 (5.4)	
Liver metastasis			<0.001
No	165 (58.3)	1663 (81.4)	
Yes	105 (37.1)	282 (13.8)	
Unspecific	13 (4.6)	98 (4.8)	
Lung metastasis			0.616
No	236 (83.4)	1654 (81.0)	
Yes	33 (11.7)	275 (13.5)	
Unspecific	14 (4.9)	114 (5.6)	
Surgery			0.067
No	256 (90.5)	1781 (87.2)	
Yes	27 (9.5)	230 (11.3)	
Unspecific	0	32 (1.6)	
Chemotherapy			<0.001
No	79 (27.9)	822 (40.2)	
Yes	204 (72.1)	1221 (59.8)	
Radiotherapy			<0.001
No	165 (58.3)	783 (38.3)	
Yes	118 (41.7)	1260 (61.7)	
Survival			0.124
2‐year disease specific survival	18.2%	23.4%	
5‐year disease specific survival	9.3%	14.5%	

At the time of diagnosis, most ENEC patients were AJCC 7th stage IV (48.1%), and only 4.6% were stage I. Poorly differentiated ESCC also showed high aggressiveness, with 33.7% of stage IV patients and 27.3% of stage III patients. Metastasis occurred in more than half of the ENEC patients (53.4%), which was significantly higher than poorly differentiated ESCC patients (33.7%, *p* < 0.001). The most common metastatic site for both ENECs (37.1%) and poorly differentiated ESCC (13.8%) was the liver. Bone (13.1%) and brain (5.3%) metastasis were more common in ENECs, while lung metastasis showed no difference (*p* = 0.616) in two groups.

**FIGURE 1:**
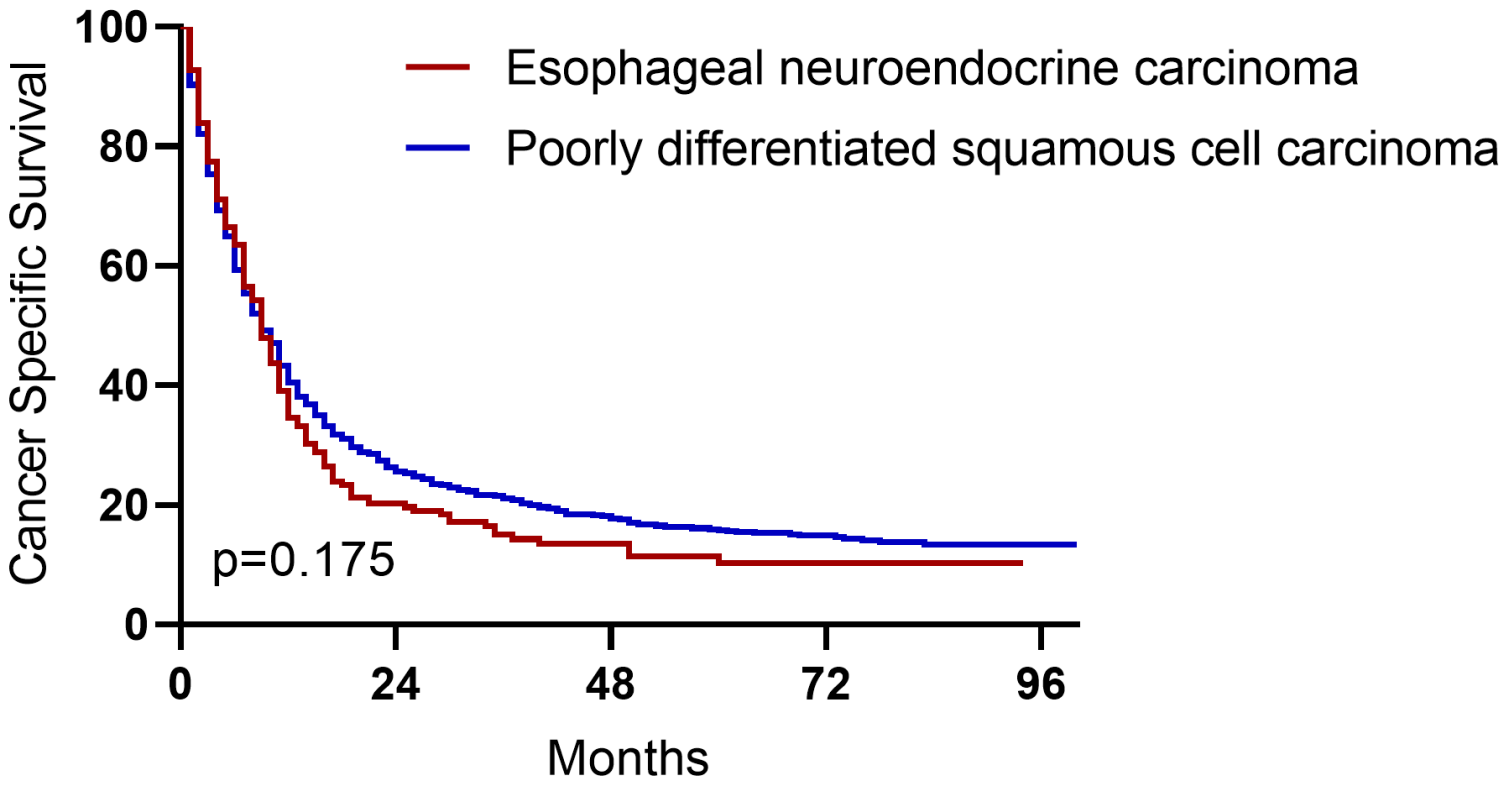
Cancer‐specific survival of esophageal neuroendocrine carcinoma and poorly differentiated esophageal squamous cell carcinoma patients

As for treatment, 90.5% ENEC patients and 87.2% poorly differentiated ESCC patients received surgery. 72.9% ENEC patients received chemotherapy, and 41.7% of them received radiotherapy. However, as shown in Figure 1, despite receiving the above treatments, survival of both ENEC and poorly differentiated ESCC were both disappointing, and did not differ significantly (*p* = 0.124).

### 3.3 Patient survival analysis

The 2‐ and 5‐years disease‐specific survival rates of ENEC were 18.2% and 9.3%, respectively. Results of univariate and multivariate analyses of the ENEC cohort are shown in Table 3. According to the univariate analysis, “M1” stage (*p* < 0.001), “Bone metastasis” (*p* = 0.003), “Brain metastasis” (*p* = 0.001), “Liver metastasis” (*p* < 0.001), and “Lung metastasis” (*p* = 0.001) were significantly associated with poor survival. Of note, tumor located in the middle third of the esophagus showed a possible trend toward significance (*p* = 0.050) in poor prognosis. In addition, “Surgery” (*p* < 0.001), “Chemotherapy” (*p* < 0.001), and “Radiotherapy” (*p* < 0.001) all led to improved survival.

**TABLE 3:** Univariate and multivariate COX analysis for esophageal neuroendocrine carcinoma patients

Clinical characteristics	Univariate	*p* value	Multivariate	p value
	HR (95%CI)		HR (95%CI)	
Sex				
Male	1		—	—
Female	1.070 (0.790–1.450)	0.660	—	—
Age				
<65	1		—	—
≥65	1.036 (0.794–1.351)	0.797	—	—
Race				
White	1		—	—
Black	0.929 (0.565–1.529)	0.773	—	—
Other	0.758 (0.472–1.216)	0.251	—	—
Marital status				
Married	1		—	—
Singled	1.033 (0.782–1.365)	0.819	—	—
Location				
Upper third of esophagus	1		1	
Middle third of esophagus	1.825 (1.000–3.329)	0.050	2.215 (1.183–4.145)	0.013
Lower third of esophagus	1.468 (0.844–2.554)	0.174	1.643 (0.923–2.927)	0.092
T‐stage				
T1	1		—	—
T2	0.838 (0.454–1.548)	0.573	—	—
T3	0.635 (0.407–0.992)	0.046	—	—
T4	1.237 (0.784–1.950)	0.361	—	—
N‐stage				
N0	1		—	—
N1	1.098 (0.793–1.521)	0.573	—	—
N2	0.950 (0.528–1.711)	0.865	—	—
N3	1.439 (0.683–3.031)	0.338	—	—
M‐stage				
M0	1		—	—
M1	2.582 (1.941–3.434)	<0.001	—	—
Bone metastasis				
No	1		—	—
Yes	1.742 (1.209–2.511)	0.003	—	—
Brain metastasis				
No	1		1	
Yes	2.463 (1.445–4.201)	0.001	1.942 (1.113–3.390)	0.019
Liver metastasis				
No	1		1	
Yes	2.159 (1.634–2.851)	<0.001	2.028 (1.517–2.713)	<0.001
Lung metastasis				
No	1		—	—
Yes	1.950 (1.303–2.917)	0.001	—	—
Surgery				
No	1		1	
Yes	0.281 (0.156–0.505)	<0.001	0.396 (0.216–0.726)	0.003
Chemotherapy				
No	1		1	
Yes	0.468 (0.351–0.625)	<0.001	0.388 (0.282–0.533)	<0.001
Radiotherapy				
No	1		—	—
Yes	0.527 (0.401–0.694)	<0.001	—	—

**FIGURE 2:**
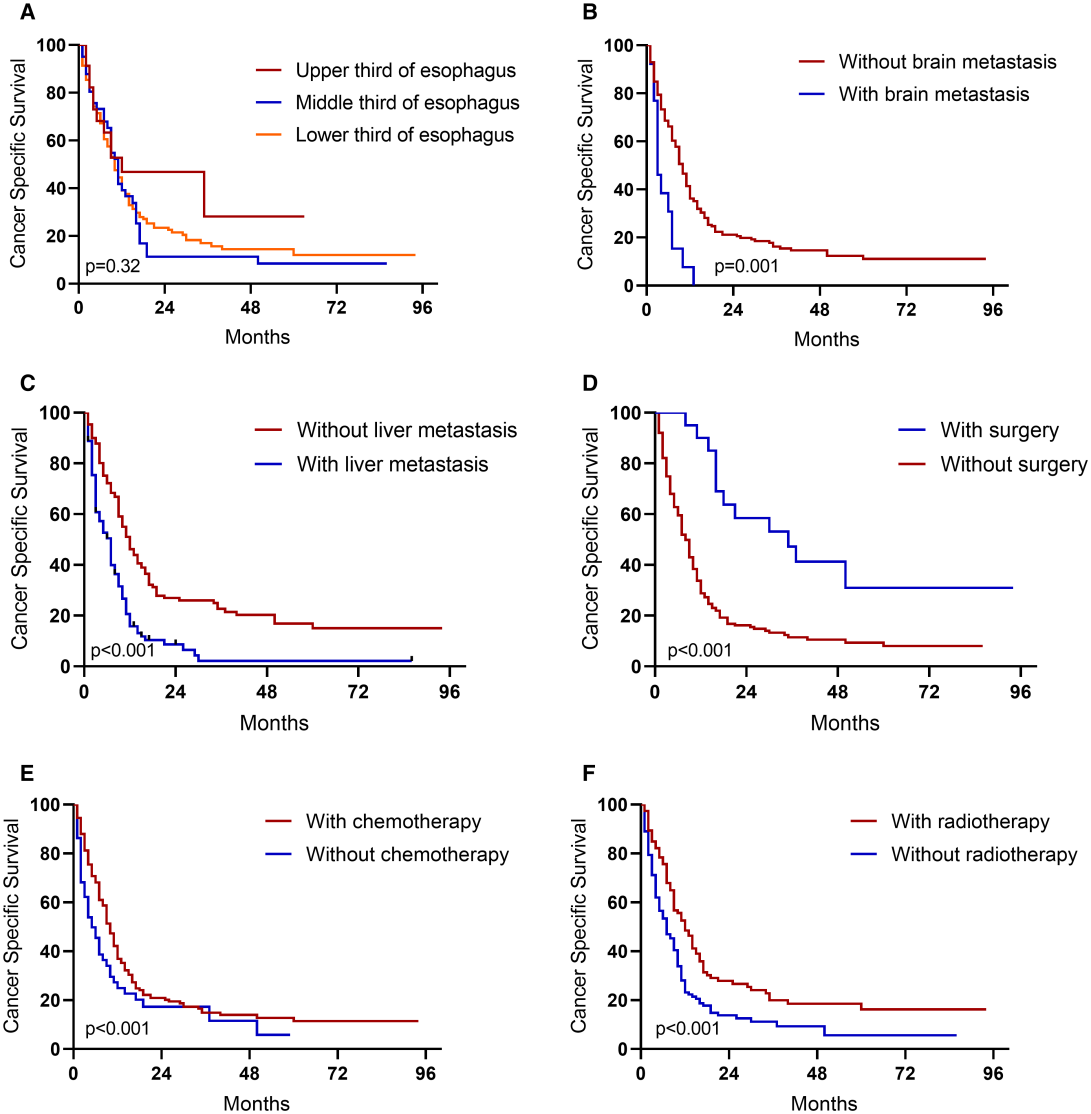
Cancer‐specific survival of esophageal neuroendocrine carcinoma patients. A. Different tumor locations. B. With or without brain metastasis. C. With or without liver metastasis. D. With or without surgery. E. with or without chemotherapy. F. with or without radiotherapy

On multivariate analysis, tumor located in the middle third of the esophagus (*p* = 0.013), “Brain metastasis” (*p* = 0.019), and “Liver metastasis” (*p* < 0.001) were independent predictors of worse outcomes. “Surgery” (*p* = 0.003), and “Chemotherapy” (*p* < 0.001) were associated with better survival. Corresponding Kaplan–Meier curves are shown in Figure 2.

### 3.4 Discussion

ENEC is a rare, aggressive, and highly metastatic disease with poor prognosis. In this study, we obtained data from 283 ENEC patients, analyzed their basic characteristics, treatment and survival. Considering the rarity of this disease, our study was a large‐scale study which provided valuable analysis of prognostic related factors. Our study also showed that different subtypes of ENEC possess similar clinical features, and we were the first study to demonstrate the similarities and differences in clinicopathological features between ENEC and poorly differentiated ESCC.

As mentioned above, ENEC can be divided into small‐ and large‐cell NEC according to histological features. Many previous studies focused only on esophageal small cell carcinoma, leading to a lack of understanding about large‐cell ENEC. According to our study, large‐cell ENEC were extremely rare, accounting for only 11.3% of all ENECs. Interestingly, our study showed no significant difference in most clinical features, as well as cancer‐specific survival, among the two subtypes. Therefore, we believe that since large‐cell ENEC is extremely rare, therapeutic strategies for esophageal small cell NEC could also be applied in esophageal large cell NEC.

Squamous cell carcinoma is another aggressive malignancy of the esophagus. Among all ESCCs, the poorly differentiated ESCC share some histological similarities with ENEC, making it difficult to distinguish between the two. However, accurate pathological diagnosis of ENEC is critical for patient management. Our study found that the lower third of the esophagus was the most common location of ENEC, while squamous cell carcinoma was more common in the middle third. Thus, when encountering lesions in the lower third of the esophagus, especially submucosal lesions, clinicians should be aware of the existence of ENEC. Of note, neuroendocrine neoplasms (NENs) originated from the gastroesophageal junction (GEJ) should also be considered when we discuss malignancies from the lower third of the esophagus. Zhang et al. reviewed 297 cases of GEJ‐NENs from Chinese hospitals and 274 cases from the SEER database and found that GEJ‐NENs also exhibit biological behaviors similar to those of esophageal NENs, including high rates of lymph node and distant organ metastasis, as well as poor prognosis.^15^

Previous studies have reported differences in metastatic patterns between ENEC and ESCC.^16^ Our study, focusing on the subgroup of poorly differentiated ESCC, also showed that only 33.7% of the poorly differentiated ESCC patients experienced distant metastasis, while distant metastasis occurred in 53.4% of the ENEC patients at the time of diagnosis. The previous study pointed out that ESCC patients can benefit from primary site surgery even with distant metastases, while ENEC patients only benefit from surgery when the disease is limited.^16^ Since ENEC is still classified according to the TNM staging of ESCC, we also agree that a unique staging and grading system for ENECs is needed to guide clinical decisions.

On the other hand, it is difficult to distinguish ENEC from poorly differentiated ESCC because synchronous development of NEC and SCC is also common. A serial histological examination of 42 ENEC specimens revealed in situ involvement of squamous cell carcinoma in 50% of the cases.^17^ The squamous cell component often overlie NECs, leading to high rates of misdiagnosis.^17^ There have also been cases reporting that squamous cell carcinoma could even convert into NEC after chemoradiotherapy,^18^ and studies in esophageal adenocarcinoma revealed that neuroendocrine differentiation was significantly related to resistant to chemoradiotherapy.^19^ Considering their difference in treatment, more attention should be paid to improve the accuracy of initial diagnosis.

In our study, the median survival time of ENEC patients was 8 months, which happened to be close to the shortest survival time reported in previous studies (8.0–28.5 months).^20^ Survival analysis showed that tumor location, distant metastasis to the brain or liver was related to poor survival. Similar to Xu's study,^21^ our study showed that patients with tumors located in the middle third of the esophagus had the worst prognosis, but fortunately, only a small portion (18.4%) of the patients had tumors located at this site. Distant metastasis was significantly related to poor prognosis, however, more than half of the patients (53.4%) experienced metastasis at the time of diagnosis. This also emphasizes the importance of screening and early diagnosis.

Surgical treatment, chemotherapy, and radiotherapy have been applied alone or in combination to improve survival in ENEC patients. However, due to the rarity of this disease, no standard treatment strategy has yet been established for ENEC.^22^ Our study showed that both surgery and chemotherapy could significantly increase cancer‐specific survival of ENEC patients. According to previous studies, many centers recommend that chemotherapy should be the cornerstone of the treatment of ENEC.^8^, ^23^ A study from the Memorial Sloan‐Kettering Cancer Center suggested that for small cell ENEC patients treated with induction chemotherapy followed by consolidative chemoradiation can achieve long‐term survival, while the contribution of surgery was unclear.^24^ A recent nationwide study from Japan revealed that there was no significant difference in survival between the operative or non‐operative groups of stage I and II ENEC patients, and for stage III and IV ENEC, chemoradiotherapy led to significantly better prognosis.^25^ On the other hand, several studies from China and Japan reported that radical esophagectomy should be considered as the preferred treatment for limited‐stage ENECs.^11^, ^21^, ^26^ Another study analyzing ENEC cases from SEER database found that surgery + chemotherapy, as well as surgery + radiotherapy, can bring more significant benefits than chemotherapy or radiotherapy alone.^10^ However, the lack of randomized controlled trials and insufficient data made it impossible to conclude which modality should be recommended to ENEC patients. Treatment could be more individualized, based on the location of the tumor, depth of invasion, lymph node or distance metastases, general condition of patients, and also the traditions of each institution.

With the advent of screening endoscopy, more gastrointestinal NENs are diagnosed at an early stage. Of note, Yasuyuki and colleagues reported a rare case of early stage ENEC.^27^ This 80‐year old man was observed for by annual endoscopy because of an unchanged lesion on the middle third of the esophagus. Three years later, the transformation of endoscopic appearance led to en bloc resection with endoscopic submucosal dissection, and this patient was finally diagnosed with small cell type ENEC (T1b). However, he declined additional surgical resection, and then experienced rapid disease progression including lymph node and liver metastasis and died within 8 months. In our study, among the total of 61 T1 patients, 39 patients already had lymph node involvement and 32 were found with distant metastasis. These observations of early stage ENEC patients indicated that the behavior of ENEC were fairly different from other pathological subtypes of esophagus cancer, and endoscopic treatment might not be appropriate except for palliative treatment.

Our study has several limitations. First, our study was limited by the available data provided by the SEER database. For example, the SEER database does not contain sufficient pathological information to grade neuroendocrine tumors according to WHO criteria. Also, as esophageal NEC is an extremely rare disease, there is a potential of misdiagnosis among different medical centers, and pathological information is unable to be verified. Additionally, other valuable information such as serum biomarkers for neuroendocrine tumors was not provided in the SEER database. Second, detailed information about surgery type, chemotherapy drug use, and radiotherapy protocol cannot be retrieved from the SEER database. Third, the SEER database does not provide follow‐up data such as recurrence time and disease‐free survival. All these limitations should be considered in future studies.

## 4 CONCLUSION

This population‐based study based on the SEER database outlined several demographic and clinical features of ENEC. Our study showed the small‐cell and large‐cell subtypes of ENEC possess similar clinical features, which indicates that therapeutic strategies for esophageal small cell NEC could be applied in esophageal large cell NEC when no clinical studies are available yet to guide treatment decision. We were also the first study to explore the similarities and differences in clinicopathological features between ENEC and poorly differentiated ESCC. ENEC and poorly differentiated ESCC share certain histological features, but differ in tumor location and metastatic rate. Unfortunately, a significant proportion of patients with newly diagnosed ENEC presented with metastatic disease, and early stage ENECs also have aggressive behaviors. Predictors of poor survival included tumor location, brain metastasis, and liver metastasis. Yet, no standard treatment strategy has been established, but surgery and chemotherapy were related to better outcomes.

## AUTHOR CONTRIBUTIONS

Conceptualization, methodology, and writing the original draft: Chuyan Chen, Haiyi Hu. Data curation and visualization: Zhibo Zheng, Yi Yang, Wei Chen. Reviewing and editing the draft: Xinwei Qiao. Conceptualization and reviewing the manuscript: Peng Li, Shutian Zhang. All authors reviewed and approved the final manuscript.

## CONFLICT OF INTEREST

The authors declare no conflict of interest.

## ETHICS APPROVAL STATEMENT

The SEER database is a publicly available database which erased personal information of all involved patients, thus informed consent from patients and approval by the institutional review board were exempted.

## Data Availability

Data used in this study were derived from the publicly avaliable SEER database (https://seer.cancer.gov/).
